# Modular Approaches to Synthesize Activity- and Affinity-Based Chemical Probes

**DOI:** 10.3389/fchem.2021.644811

**Published:** 2021-04-15

**Authors:** Antonie J. van der Zouwen, Martin D. Witte

**Affiliations:** Chemical Biology II, Stratingh Institute for Chemistry, University of Groningen, Groningen, Netherlands

**Keywords:** chemical probe design, activity-based probe, affinity-based probe, protein profiling, DNA-templated, SuFEx, iminoboronate chemistry

## Abstract

Combinatorial and modular methods to synthesize small molecule modulators of protein activity have proven to be powerful tools in the development of new drug-like molecules. Over the past decade, these methodologies have been adapted toward utilization in the development of activity- and affinity-based chemical probes, as well as in chemoproteomic profiling. In this review, we will discuss how methods like multicomponent reactions, DNA-encoded libraries, phage displays, and others provide new ways to rapidly screen novel chemical probes against proteins of interest.

## Introduction

**Figure 1:**
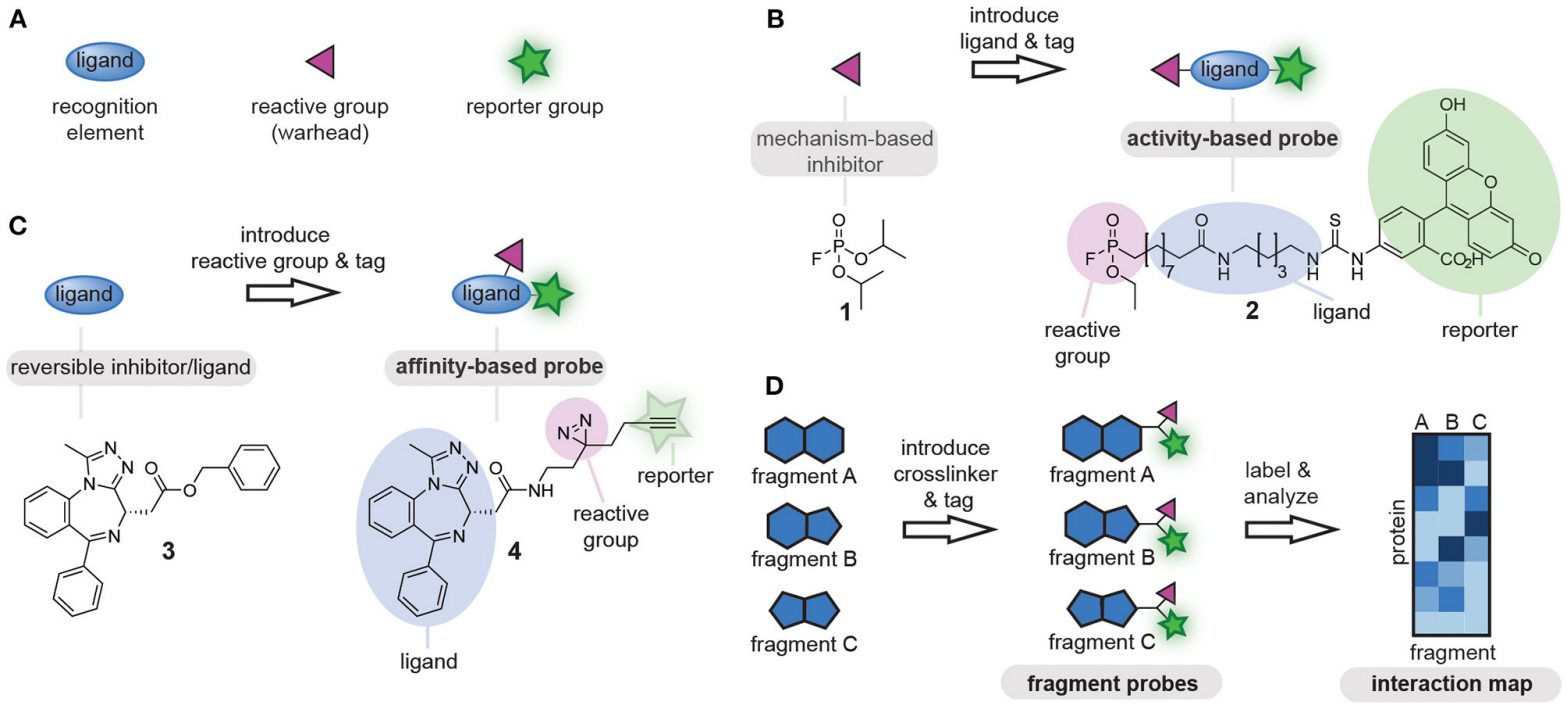
**(A)** Schematic representations of the components of chemical probes. **(B)** Schematic representation of activity-based chemical probe design. The reactive group is leading in the design and commonly reactive groups that exploit the mechanism of the target enzyme are used as starting point. **(C)** Schematic representation of affinity-based chemical probe design. The ligand is leading in the design of affinity-based probes and often known bioactive molecules are used as starting point. **(D)** Schematic representation of a chemoproteomic fragment library. Small molecular fragments (ligands) are equipped with the identical reactive groups and reporter groups and are screened against complex protein mixtures using chemoproteomic approaches. To visualize the differences between the fragments, the proteins that bind to different fragments are plotted in an interaction map. The color correlates with the enrichment fold. Dark colored proteins are enriched the most. The resulting selectivity profiles enable the identification of new probe-protein combinations.

Chemical tools that covalently modify proteins are of great interest for fundamental biological research as well as for biomedical applications. Known as chemical probes, these chemical tools have been utilized for the study of proteins, the identification of protein-natural product interactions and for the screening of new drug leads, among others (Benns et al., 2021). Fully functional chemical probes contain at least the following three elements: (1) a ligand, to confer selectivity toward the proteins of interest, (2) a reporter group (e.g., fluorophore, affinity tag, or bioorthogonal tag for later functionalization) (Speers and Cravatt, 2004), and (3) a reactive group that covalently reacts with the proteins of interest (Figure 1A) (DeGruyter et al., 2017). Which of these probe parts is leading in the probe design is determined by the protein target and dependent on the biological question that is being addressed.

Rational design of chemical probes for proteins of interest is typically guided by either the reactive group or the ligand. The reactive group often serves as starting point for the development of probes for enzymes that form a covalent intermediate with their substrate. These so-called activity-based probes exploit the mechanism of the target enzyme to form a covalent probe-protein adduct, and can be obtained by equipping an appropriate mechanism-based reactive group with a ligand and a tag (Figure 1B). The activity of a whole enzyme family can be profiled with probes that contain minimalistic ligands, as the reactive group will confer selectivity toward the enzyme family. One of the first examples of this approach was the conversion of fluorophosphate **1**, which inhibits serine hydrolases, into fluorophosphonate probe **2**. This fluorophosphonate probe is nowadays commonly used for profiling of the serine hydrolases family (Liu et al., 1999). If the goal is to target a single enzyme within an enzyme family, selectivity should be generated toward the target enzyme by tuning the ligand part of the probe. This is commonly done when profiling specific proteases by incorporating substituents that target the substrate binding pockets (Greenbaum et al., 2002; Berger et al., 2006; Screen et al., 2010; Geurink et al., 2013; de Bruin et al., 2016; van de Plassche et al., 2020).

For proteins and enzymes that do not form covalent intermediates with their ligands and substrates, the design of the chemical probe often starts from the ligand part of the probe. In this method, a ligand of interest is equipped with a reporter group and a reactive group. The latter is responsible for crosslinking the probe to the protein of interest (Figure 1C). This second method is very suitable to convert known binders of proteins of interest into affinity-based probes, and has, for example, been used to convert bromodomain inhibitor **3** into probe **4** (Li Z. et al., 2014). Furthermore, it can also be used to identify the targets of natural products. A large variety of reactive groups has been reported over the years, ranging from chemical crosslinkers that react specifically with a single type of amino acid residue to photocrosslinkers that form highly reactive carbene, nitrene or bi-radical species under UV-irradiation and that react with any amino acid residue in proximity of the probe-binding pocket (DeGruyter et al., 2017; Murale et al., 2017). The choice for a reactive group in this case is not so clear cut as compared to the design of activity-based probes. To assure efficient crosslinking, the reactive group has to be positioned near a suitable amino acid. This requires knowledge of the three dimensional-structure of the target protein. In absence of structural information, or when the target proteins are unknown, several derivatives with different reactive groups and different positioning of the reactive group on the ligand scaffold will have to be synthesized, until a probe is obtained with the desired target engagement and selectivity profile (Wright et al., 2017).

In the past decade, another method has become popular in probe development, namely the chemoproteomic profiling of ligandable proteins (Backus et al., 2016; Parker et al., 2017). In this method, a library of small molecular fragments that contain a reactive group is prepared (Figure 1D). These libraries can be designed in two different ways. One way is to introduce the reactive group on fragments that contain a bioorthogonal tag. Libraries of this kind can be screened directly, as the bioorthogonal tag can be functionalized for the purpose of visualization or affinity enrichment (Parker et al., 2017). Analysis of the modified proteins by mass spectrometry can reveal a pattern of selectivity for the different fragments and potentially uncover covalent modifiers of proteins that have not been targeted before. In the second type of libraries, the bioorthogonal handle is omitted in the fragments themselves. These libraries are analyzed by competition experiments with a broad-spectrum residue specific probe. Using this method, ligandable cysteine (Backus et al., 2016), lysine (Hacker et al., 2017), carboxylic acid (Cheng et al., 2017; Bach et al., 2020; Ma et al., 2020), tyrosine (Hahm et al., 2020), and methionine (Lin et al., 2017) residues have been identified. The latter chemoproteomic method provides probe leads rather than fully functional chemical probes, and these leads still have to be converted into fully functional probes by introducing a suitable reporter group as described for the second method.

Irrespective of the method that is being used, chemical probes contain the aforementioned ligand, reactive group, and reporter group. Combining these three elements in a single probe can take significant synthetic effort, especially when several variants of the probe have to be synthesized in order to identify the most efficient probe. To simplify the synthesis, modular approaches have been developed in which the activity- and affinity-based probes are prepared from different probe fragments. These modular approaches make the synthesis of probes less time-consuming. Different versions of the probes can be prepared by replacing one of the modalities for one that has different properties. Examples hereof would be exchanging a diazirine photocrosslinker for a sulfonyl fluoride crosslinker, or a fluorescent reporter group for a bioorthogonal handle. The relative ease by which these elements can be interchanged in modular approaches makes these approaches excellent tools to screen different ligand-reactive group combinations.

Modular approaches are commonly employed when probes are prepared by solid phase synthesis (Berger et al., 2006). Activity-based probes for various proteases have been optimized by synthesizing a library of peptides and functionalizing these with a reactive group and a reporter. Recently, a similar approach was used by the Verhelst lab to synthesize probe libraries for serine hydrolases (Vanhoutte et al., 2020). In this case, different reactive groups were incorporated in the final steps of the solid phase synthesis.

In contrast, linking fragments in solution has long been considered to be more challenging. For efficient synthesis of a probe library, the chemistry that is used to couple the ligand to the reactive group and the reporter group should be robust and high-yielding, and the number of purification steps should be minimal. Over the past decade, multicomponent reactions, click reactions, DNA-templated reactions, macrocyclization of peptides, and trifunctional reagents have been reported that allow modular synthesis of chemical probes in solution. In this review, we will discuss the advantages and disadvantages of these approaches. We will focus on modular approaches that allow the synthesis of probes *in situ* prior to the screening step, as screening of reaction mixtures simplifies the identification of probes for proteins of interest.

### Solution Phase Combinatorial Synthesis of Probes

Combinatorial synthesis approaches that yield a structurally diverse panel of probes in a minimal number of steps increase the chances of identifying a small molecule probe for a protein of interest. To be compatible with solution phase synthesis, the synthesis scheme ideally should use readily available or easy to synthesize building blocks, should enable straightforward diversification of the scaffold and should contain a limited number of purification steps. The first step toward the development of a combinatorial probe synthesis approach that fulfills these requirements was reported in 2012 by the group of Cravatt (Cisar and Cravatt, 2012). Using multicomponent reactions (MCRs) and cross-coupling reactions, they synthesized a small library of photocrosslinker probes around 5-benzoyl indole (BzIndole) and 7-benzoyl-benzo-1,4-diazepin-2,5-dione (BzBD) scaffolds. These chemistries were selected, as MCRs enable the synthesis of complex molecular scaffolds from at least three different building blocks in a single step and cross-coupling reactions allow straightforward further diversification. Analysis of the biological activity of the BzBD and BzIndole libraries by gel-based protein profiling and by chemoproteomics revealed that different members of the probe library labeled different subsets of proteins. This illustrates the power of the approach, but a broad applicability was somewhat impaired by the still complicated multistep synthesis of the fully functional probes.

**Figure 2:**
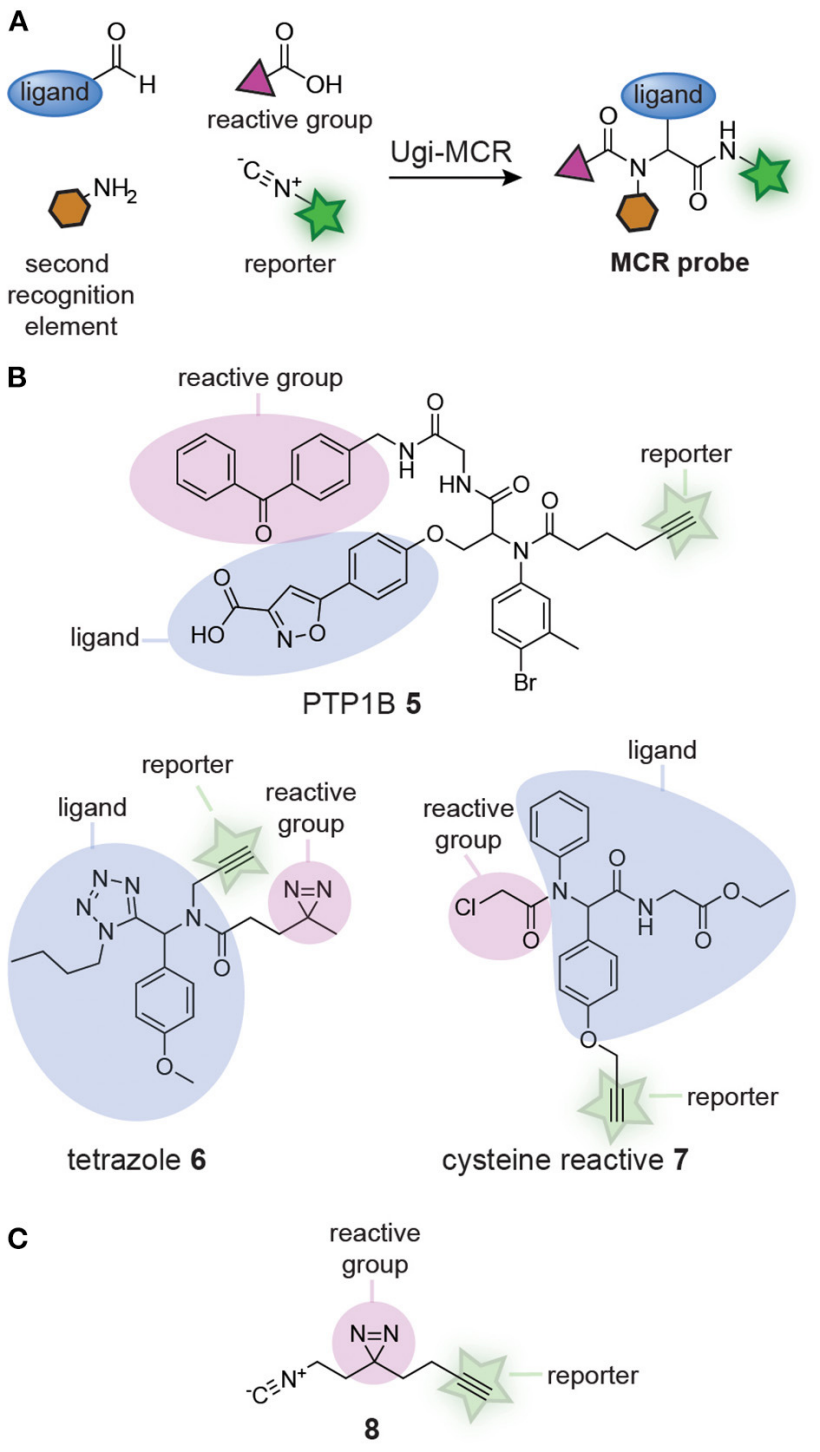
**(A)** Schematic representation of probe synthesis by multicomponent reactions (MCRs). Ligands can be combined with reactive groups and reporter groups to generate probes in a single step. **(B)** Examples of probes discovered by screening probe libraries prepared via MCRs. **(C)** Structure of the isocyanide building block (**8**), containing both a diazirine reactive group and an alkyne reporter group, developed by Jackson and Lapinsky, that can be used for the synthesis of MCR probes.

Several groups realized that affinity-based probe libraries could be prepared in a single step by adapting the design of the MCRs (Figure 2A). It was reasoned that different building blocks, each containing one of the structural elements of a probe (i.e., a reactive group, a bioorthogonal handle or a ligand), could be combined in the MCR. Each combination of the building blocks should yield a unique affinity-based probe with a distinct biological activity. In 2012, the group of S. Yao demonstrated the feasibility of this approach (Ge et al., 2012). They synthesized probes for the phosphotyrosine phosphatases PTP1B and MptpB with an Ugi MCR. An aldehyde-containing isoxazole derivative that mimicked phosphotyrosine, thus functioning as the protein-binding ligand, a benzophenone isocyanide photocrosslinker, and a hexynoic acid reporter group were reacted with 25 structurally different, commercially available amines. The Ugi MCR yielded fully functional probes in a single step (see compound **5**, Figure 2B). The binding affinity of the resulting probes for PTP1B were in the same range as a known inhibitor of PTP1B. Labeling experiments with the probes revealed that each probe had a different labeling efficiency, demonstrating that the approach may be used to identify the most suitable substitution pattern on a probe. After this seminal paper, the group of Cravatt used the Ugi-azide MCR to prepare a 60-member library of 1,5-substituted tetrazole affinity-based probes from aldehydes, amines, isocyanides and trimethylsilyl azide (Kambe et al., 2014). Each of the reactions contained a building block with a diazirine photocrosslinker and a building block with an alkyne reporter group, thereby assuring that fully functional probes were obtained from the MCR. For an example of the obtained tetrazole probes, see structure **6** (Figure 2B). Protein-profiling with the resulting tetrazole probes showed that many of the library members labeled unique and distinct subsets of the proteome. Analysis of the targets by chemoproteomic methods identified 24 proteins that were labeled by a subset of the probes. The large diversity in the targets and the differences between the probes further illustrates the effectiveness of the MCR approach. The Ugi MCR has recently been applied to prepare cysteine reactive probes by using building blocks that contain an acrylamide or a chloroacetamide reactive group (Liu et al., 2019). Out of the 61 library members, 37 contained a bioorthogonal handle. For an example of a probe obtained from this reaction, see compound **7** (Figure 2B). Several of the probes displayed anti-proliferative activity in the prostate cancer cell line BxPC3 and chemoproteomic analysis revealed that the relatively small library already led to the identification of interesting probe leads.

In the libraries described thus far, the photocrosslinker and the reporter group were positioned on different building blocks. This restricts the diversity of the building blocks that can be used in the library preparation, since at least one component should contain the reporter group and another the crosslinker. Jackson and Lapinsky reasoned that a building block that contains both moieties would enable the synthesis of a larger number of probes by MCR (Jackson and Lapinsky, 2018). Since many MCRs use isocyanides as one of the building blocks, Jackson and Lapinsky prepared an isocyanide equipped with a diazirine and alkyne (**8**) (Figure 2C). Isocyanide **8** was successfully applied in the Passerini reaction, the Ugi 4-component reaction, the Ugi azide reaction, the Ugi 5-center-4-component reaction and the Ugi β-lactam-3-component reaction. The resulting MCR products could be readily derivatized. Reagents like the one developed by Jackson and Lapinsky should therefore simplify MCR probe synthesis even further.

**Figure 3:**
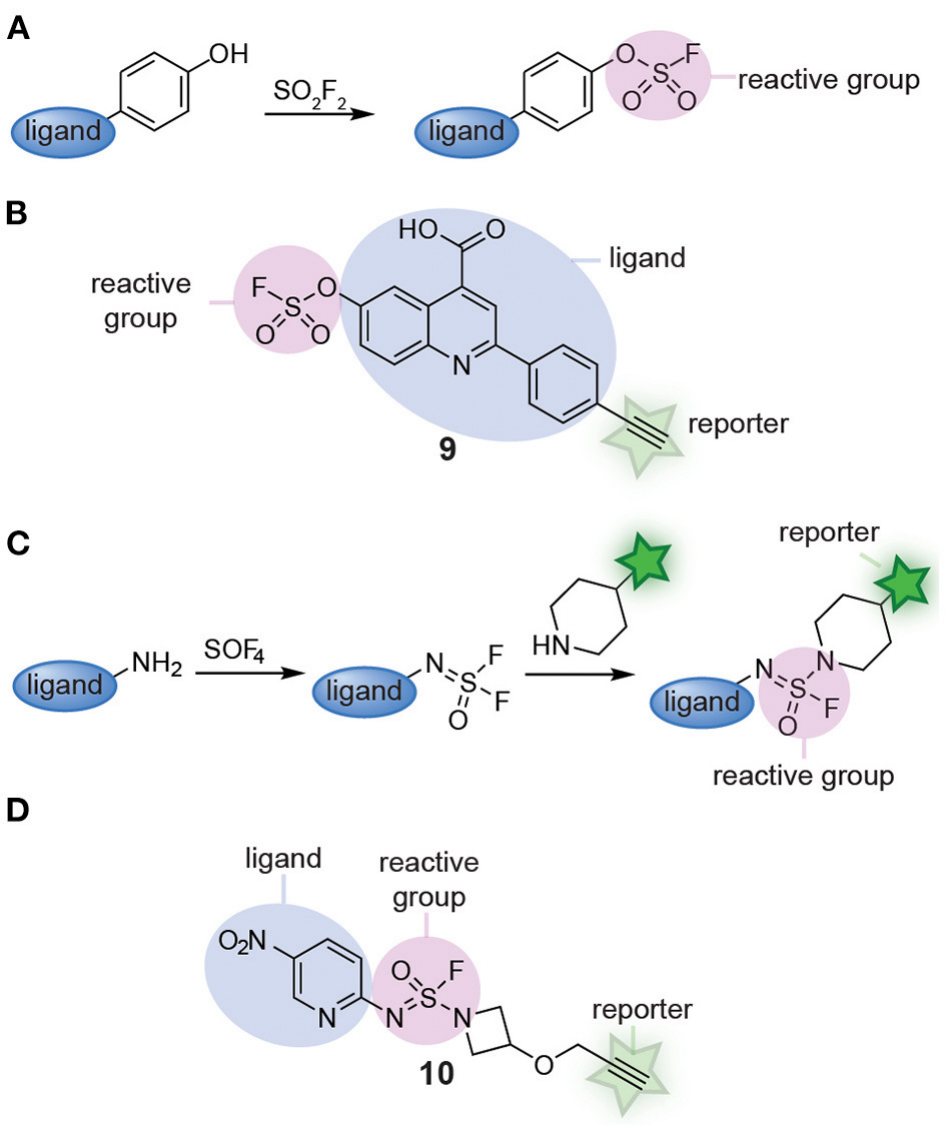
**(A)** Schematic representation of fluorosulfate probe synthesis with SuFEx. **(B)** An example of a fluorosulfate probe (**9**) prepared from a phenolic compound via SuFEx chemistry. **(C)** Schematic representation of sulfuramidimidoyl fluoride probe synthesis with SuFEx. **(D)** An example of a sulfuramidimidoyl fluoride probe (**10**) prepared from a primary and a secondary amine.

Recently, Sulfur(VI) fluoride exchange (SuFEx) click chemistry started to be explored as an alternative for multicomponent reactions. SuFEx allows straightforward synthesis of scaffolds equipped with a sulfonyl fluoride reactive group (Jones and Kelly, 2020). At the same time, the products of SuFEx reactions are stable enough that they can be modified further and/or act as latent reactive groups in chemical probes. The groups of Sharpless and Kelly reported a modular SuFEx approach for the synthesis of probes. By reacting phenolic compounds with sulfuryl fluoride, they prepared several fluorosulfate probes (Figure 3A). Minimalistic versions of the probe reacted with lipid-binding proteins selectively (Chen et al., 2016). In a follow-up paper, the groups of Kelly, Sharpless, and Wilson converted one quinolone scaffold (compound **9**, Figure 3B) and two imidazole scaffolds into fluorosulfate probes. By equipping the scaffold with a reactive group and a reporter group, the probes could be screened against the complete proteome and the targets of the drug-like scaffolds could be identified by chemoproteomic methods. Gel-based protein profiling and chemoproteomic analysis revealed that the resulting probes label different targets than the minimalistic probes (Mortenson et al., 2018). Extending on this work, the groups of Sharpless and Kelly very recently applied a different kind of SuFEx reaction to prepare sulfuramidimidoyl fluoride probes (Figure 3C). They discovered that thionyl tetrafluoride (SOF_4_) reacts selectively with primary amines to form iminosulfur oxydifluoride products and that a second fluoride can be substituted with secondary amines or aryl silyl ethers in follow-up steps to form sulfuramidimidoyl fluoride and sulfurofluoridoimidate products (Li et al., 2017). With this modular two-step approach, a structurally highly diverse library of 16 fully functional sulfuramidimidoyl fluoride probes was prepared (Brighty et al., 2020). Compound **10** depicts an example of a probe prepared via this method (Figure 3D). From this relatively small library, novel probes for macrophage migration inhibitory factor, PARP1, and PARP2 were identified. These results clearly illustrate the strength of modular probe synthesis by SuFEx.

In short, major advances have been made in the solution-phase combinatorial synthesis of small molecule probes over the past decade. The application of multicomponent reactions and SuFEx click reactions has provided rapid access to structurally diverse probes. The recently reported multifunctional building blocks (see also section modular synthesis of small molecule chemical probes) will enable the synthesis of libraries with even larger diversity. Moreover, the relative stability of SuFEx reagents should enable combining SuFEx strategies with MCRs, which will increase the diversity in the scaffolds further. We reason that the applicability of these approaches can be extended even further by developing MCR and SuFEx reactions that allow direct screening of the reaction mixtures and thereby increase throughput. Based on the clean nature of SuFEx reactions, direct screening of the library may be feasible (Kitamura et al., 2020).

### DNA-Templated Probe Synthesis

**Figure 4:**
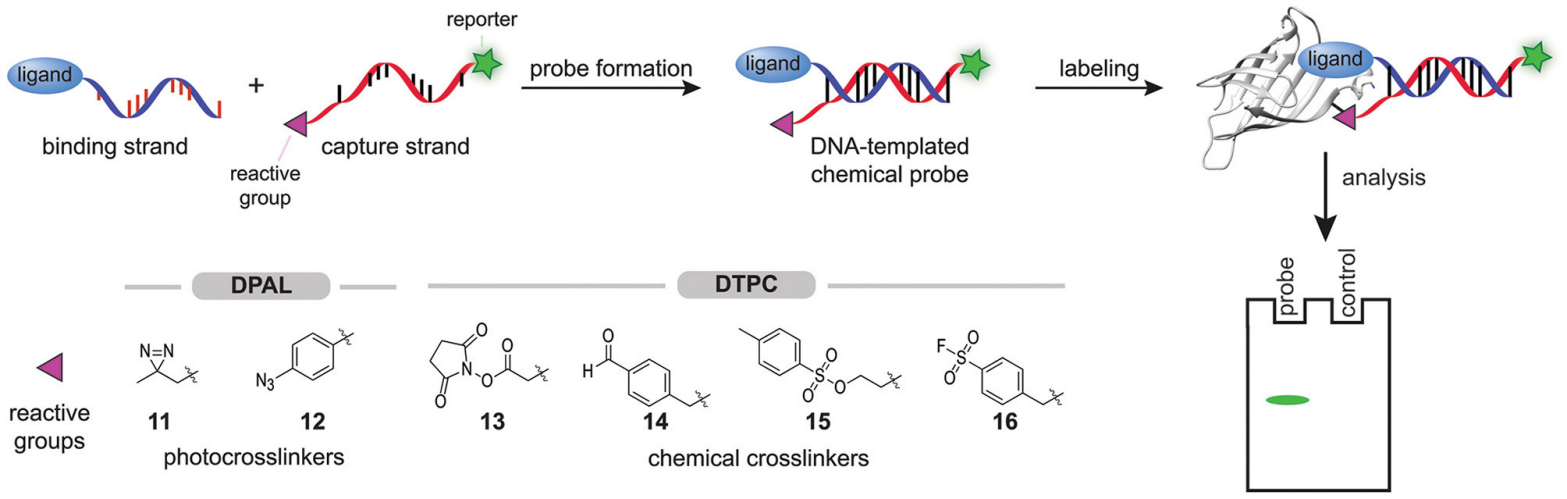
Schematic representation of the modular synthesis of chemical probes by DNA-technology. A binding strand, functionalized with a ligand that binds selectively to the protein of interest, is hybridized with a capture strand that contains a protein reactive group. In DPAL, the 3′ of the capture strand is functionalized with one of the depicted photocrosslinkers and the 5′-end of the capture strand contains a reporter group. In DTPC, the 3′ of the capture strand is functionalized with one of the depicted chemical crosslinkers and the 5′-end of the capture strand contains a reporter group. Upon binding of the ligand, the reactive group on the capture strand will covalently modify the protein of interest. The labeled proteins can be visualized via the reporter group on the capture strand.

In 2013, Li X. et al. started exploring the feasibility of modular probe synthesis using DNA technology (Li G. et al., 2013). They hypothesized that a binding strand—a single-stranded DNA functionalized with a ligand known to bind to the protein of interest—could be hybridized with a capture strand—a complementary DNA-strand that is equipped with a reporter group and a photocrosslinker—to form affinity-based probes (Figure 4). For the proof-of-concept studies, they coupled several sulfonamide ligands, desthiobiotin, or chymostatin to the 5′-end of the binding strand. They introduced a diazirine group (**11**) at the 3′-end of the capture strand to assure that the photocrosslinker, after hybridization and binding of the probe to the target protein, is positioned in the proximity of the protein. The 5′-end of the capture strand was coupled to a fluorescein fluorophore to detect the covalent protein-probe adducts formed upon irradiation with UV-light. Labeling experiments with these DNA-templated probes on carbonic anhydrase II, avidin and chymotrypsin proved that affinity-based probes could be formed in this way. Fluorescent labeling of the target protein was observed for all ligand-protein pairs tested. Control experiments with DNA strands in which the protein binding ligand was omitted, with capture strands with a mismatched DNA-sequence, or with denatured target proteins did not result in a fluorescent signal, showing that the labeling was dependent on binding of the ligand and hybridization with the capture strand. In order to test the selectivity of the probes, samples were spiked with BSA or, in one case, with cell lysate. No background fluorescent signal was observed, indicating that the probes are selective. The labeling efficiency of the probes proved to be dependent on the hybridization site between the capture strand and the binding strand and thus, the hybridization site may be varied to optimize the positioning of the photocrosslinker.

Li and coworkers illustrated the modular nature of the approach by converting the immunophilin inhibitor AP1497 into an affinity-based probe that selectively labeled FKBP12 in Jurkat cell lysate. Depending on the capture strand that was used, the labeled FKBP12 could either be detected by in-gel fluorescence scanning or, when the capture strand contained a biotin moiety instead of a fluorophore, be enriched by pulldown experiments (Li G. et al., 2013). They dubbed their approach DNA-programmed photoaffinity labeling (DPAL).

The low labeling yield prompted Li et al. to study the effect of the photocrosslinker on the efficiency. Both the type and the number of photocrosslinkers incorporated in the capture strand determined the efficiency of DPAL. In their hands, arylazides (**12**) gave the best labeling yields and the efficiency could be increased further by using captures strands that contain multiple arylazide photocrosslinkers (Li G. et al., 2014). In the past years, DPAL has found several applications. DNA-hairpins containing a sequence that is recognized by the transcription factor P50 have been employed for the synthesis of DPAL probes that label this subunit of the NFκ-B transcription factor (Liu et al., 2015). The targets of an Aurora kinase inhibitor have been identified with DPAL (Wang et al., 2017). Probes for histone modification reader proteins have been prepared by conjugating an N-terminal histone 3-derived peptide that is trimethylated on lysine residue 4 to the binding strand (Bai et al., 2016). Very recently, the group of Li exploited DPAL labeling of endogenous membrane proteins for on cell screening of DNA-encoded chemical libraries (Huang et al., 2021).

DNA-templated probe synthesis has also been used to prepare probes that contain chemical crosslinkers. This so-called DNA-templated protein conjugation (DTPC) is analogous to DPAL, but now the complementary capture strand contains reactive groups like *N*-hydroxysuccinimide activated esters (OSu ester, **13**), aldehydes (**14**), tosylates (**15**), or sulfonyl fluorides (**16**) (Figure 4) (Rosen et al., 2014; Bai et al., 2016; Kodal et al., 2016; Wang et al., 2017; Yan et al., 2018). For example, the group of Gothelf hybridized a binding strand functionalized with a tris-nitrolotriacetic acid (NTA)-group with a capture strand equipped with OSu ester **13** to label His-tagged proteins in the presence of nickel(II) (Rosen et al., 2014). The rapid hydrolysis of the activated ester proved to be disadvantageous for long-term storage of the capture strand. In a subsequent paper, Gothelf and coworkers demonstrated that aldehydes (**14**) serve as a suitable and more stable alternative. Imine formation followed by reductive amination enabled efficient modification of the metal binding proteins, serotransferrin, alkaline phosphatase, and IgG1 antibodies (Kodal et al., 2016).

As shown, DPAL and DPTC are not only compatible with a variety of reactive groups, but also with different ligand types. Small molecule ligands, tris-NTA tags, peptides, and even aptamers—single-stranded DNA or RNA molecules that can bind protein targets—have been successfully employed as binding elements in these DNA-templated probe development approaches. This flexibility does not only give many options to target a desired protein, but also widens the targetable protein space, increasing the chance of developing a successful chemical probe.

**Figure 5:**
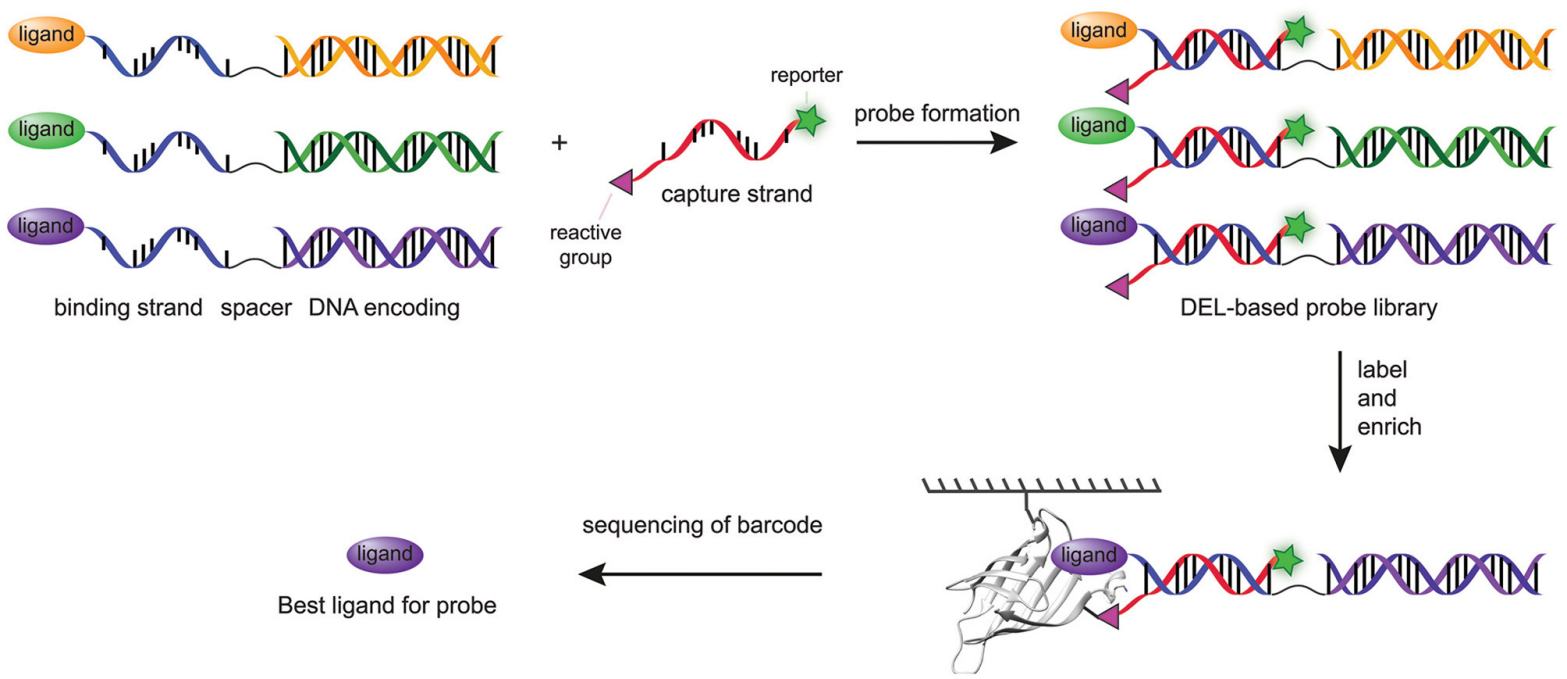
Schematic representation of chemical probes prepared with a combination of DPAL/DTPC and DEL. A DEL library is functionalized with a general ssDNA linked to a dsDNA barcode. The general strand is hybridized with the capture strand. After covalent labeling of the target protein, the target protein is enriched by affinity enrichment. The unbound probes are washed away and the bound probes are analyzed by DNA sequencing.

The most recent major step forward in the modular probes synthesis with DNA technologies was adapting DPAL and DTPC to prepare chemical probes from DNA-encoded libraries of small molecules (Denton and Krusemark, 2016). In DNA-encoded libraries (DELs), small molecules or peptides are linked to a unique double-stranded DNA (dsDNA) that encodes for the particular compound (Kleiner et al., 2011; Goodnow et al., 2017). Affinity-based enrichment of binders, often through the use of an immobilized protein target, and subsequent decoding of the DNA barcode with PCR allows the identification of the molecular structures of the binders. The abundancy of the barcode correlates with the binding affinity of the small molecule. Since DNA-sequencing technologies are able to measure the abundancies of different DNA strands within a single sample, many compounds can be screened in a single experiment (Kleiner et al., 2011). Consequently, DELs can, in contrast to more traditional high-throughput screening methods, contain many more compounds (as high as 5.1 billion) without hampering the screening process (Goodnow et al., 2017). Denton and Krusemark reasoned that the ability to combine the DNA-templated probe synthesis approaches with DELs would thus facilitate simultaneous screening of a larger number of ligand-reactive group combinations in a straightforward fashion. Key step in the preparation of DPAL and DTPC is the hybridization of the binding strand with the complementary capture strand. The dsDNA encoding sequences in DELs are not compatible with this step. Denton and Krusemark overcame this issue by conjugating the ligand to a general single-stranded DNA sequence, which was linked via a spacer to a dsDNA encoding sequence (Figure 5). In this fashion, all the probes could be paired with a single capture probe, while each ligand still had a unique encoding sequence. Capture strands functionalized with a diazirine photocrosslinker (**11**), an arylazide photocrosslinker (**12**), an OSu ester (**13**), a tosylate reactive group (**15**), or a sulfonyl fluoride (**16**) were used in the labeling experiments. All probes labeled the model protein, carbonic anhydrase II, successfully, albeit with different efficiencies. Of the reactive groups used, the OSu ester (**13**), the sulfonyl fluoride (**16**), and the diazirine photocrosslinker (**11**) showed the most intense labeling. In contrast to the work of Li and coworkers (Li G. et al., 2014), in this case the arylazide (**12**) proved to be less efficient. A subsequent DEL screen with a high-affinity ligand, a low-affinity ligand and one control compound demonstrated that covalent labeling drastically increased the sensitivity compared to traditional affinity-enrichment methods. The high-affinity and the low-affinity ligand were enriched, respectively, 17,000 and 1,700-fold over the control compound. In comparison, the traditional affinity-based enrichment led to 720-fold enrichment of the strongest binder, while the weaker binder was not enriched at all.

Recently, Krusemark et al. expanded their methodology to two 96-member peptide DELs to identify probes for the intracellular target protein Chromobox protein homolog 7 Chromodomain (CBX7-ChD) and the membrane protein δ opioid receptor (DOR) in live cells (Cai et al., 2019). The poor membrane permeability of DNA impaired intracellular delivery of the DEL probes to the cytosolic CBX7-ChD. To introduce the probes for this protein into the cells, a cyclic cell-penetrating peptide (Qian et al., 2016) had to be attached to the DNA-barcode. Tagged versions of CBX7-ChD and DOR were used to enrich the covalently bound library members. Upon sequencing of the corresponding encoding DNA, several peptides were identified with an improved binding affinity for CBX7-ChD, further underlining the potential of DEL-based probe synthesis.

In summary, DNA-templated probe synthesis approaches have proven to be a powerful means to prepare chemical probes. The modular nature of DPAL and DTPC, and the possibility to combine them with DELs enables screening of a vast number of small molecules and reactive groups on immobilized protein targets. These properties will simplify the identification of probes. Despite the high potential of DNA-templated probe synthesis methods in combination with DELs, there are some challenges that still remain to be addressed, especially those associated with using the DNA-templated probes in a cellular context. The dsDNA of the probe makes the probe cell impermeable and while cellular uptake can be enhanced with cell-penetrating peptides, the introduction of the peptide part does complicate the synthesis of the probe. Moreover, the non-native dsDNA of the probe may be degraded by DNAses or may generate an immune response. Careful selection of the cell-type may prevent an immune response, but this will not be possible in all cases (Cai et al., 2019). Consequently, it will be challenging to use a DEL probe *in vivo*. DNA-templated probes will therefore primarily find applications in cell-based studies toward cell membrane proteins and extracellular proteins, as well as *in vitro* to study biological processes. We reason that another important application for the DEL probes will be lead discovery for small molecule chemical probes. The DEL probes provide insight into which reactive group to use and how to place it. Traditional small molecule chemical probes may be obtained from DNA-templated probes by fragment linking approaches commonly used in the medicinal chemistry field.

### Modular Approaches to Synthesize Cyclic Peptide Probes

Cyclic peptides have garnered much interest as peptide-based inhibitors (White and Yudin, 2011; Yudin, 2015). Cyclization of peptides reduces their conformational freedom, which increases the rigidity of the peptide and induces conformations that more closely resemble those of natural proteins (Kessler, 1982). The induced conformations can potentially increase the surface area of contacts between a cyclic peptide and its protein target, resulting in enhanced binding affinity and selectivity. Additionally, cyclic peptides have an increased stability against proteases compared to linear peptides. Lastly, peptide cyclization has been shown to be compatible with phage display, a powerful tool to screen potential binders (Koivunen et al., 1995; Heinis et al., 2009). Due to these favorable properties, cyclic peptides recently attracted attention as a starting point for the development of chemical probes, resulting in elegant modular methods to prepare chemical probe libraries.

**Figure 6:**
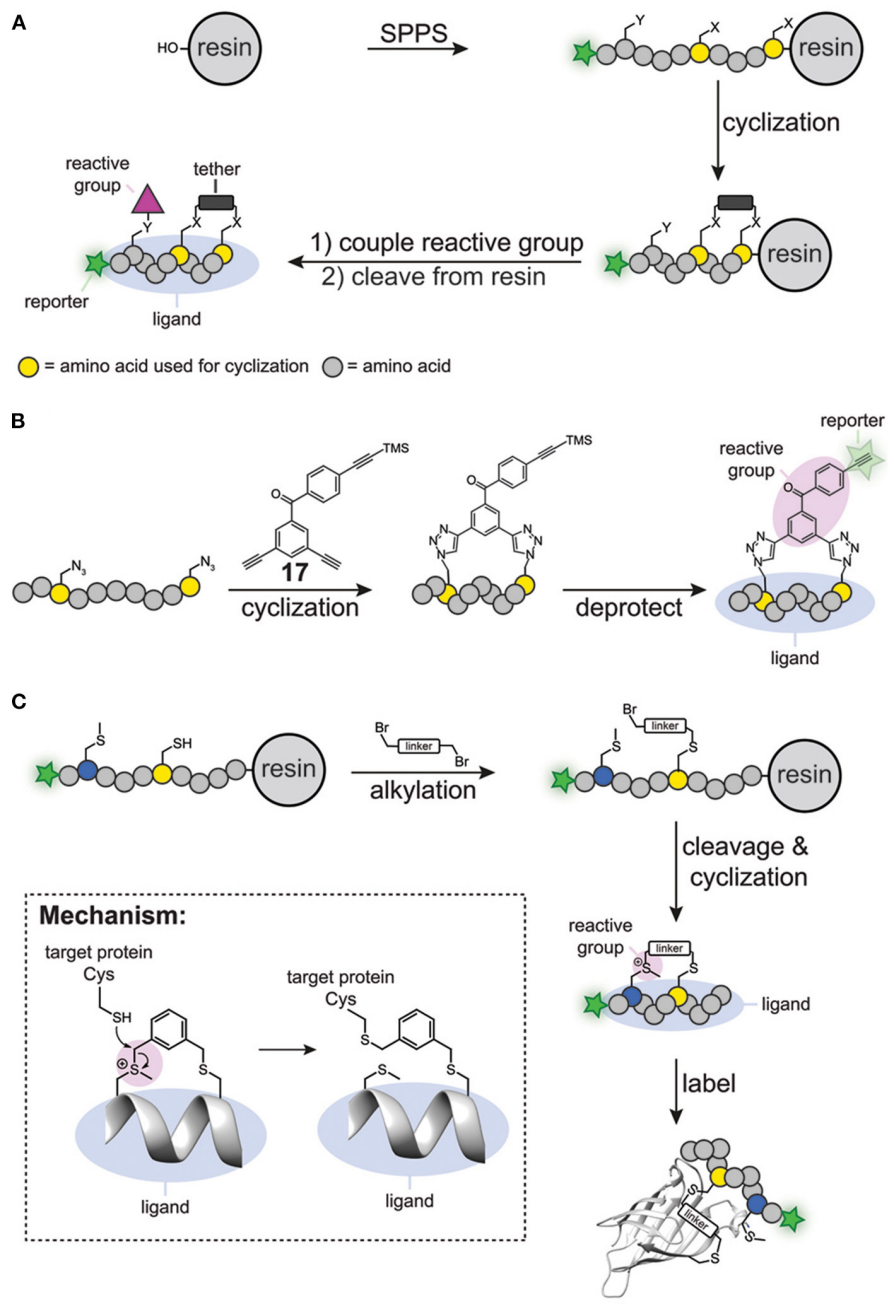
**(A)** Traditional synthesis of cyclic peptide probes. **(B)** Schematic representation of simultaneous peptide cyclisation with a clickable benzophenone photocrosslinker (**17**). **(C)** Specific alkylation of a cysteine residue and a methionine residue to prepare cyclic probes. The sulfonium reactive group reacts with cysteine residues within the proximity of the probe binding site.

The first generation of cyclic peptide probes was prepared by solid phase peptide synthesis (Figure 6A). One of the first activity-based probes containing a cyclic peptide binding element was developed for calpain-1 (Jo et al., 2012). The group of Greenbaum prepared a short synthetic peptide that contained two cysteine residues, a N-terminal epoxy succinate warhead and a C-terminal reporter group (either biotin or FITC). The peptide was cyclized by alkylating the cysteine residues with a bis(bromomethyl) alkylating agent. This process, also known as cysteine peptide stapling, induces the formation of an α-helical structure, which generates selectivity for calpain-1 over closely related cysteine proteases. Indeed, the probes efficiently labeled calpain-1 without showing any off-target activity against other cysteine proteases.

The peptide stapling approach was also used to synthesize affinity-based probes for the oncogenic proteins Bcl2A1 (de Araujo et al., 2017), BFL (Huhn et al., 2016), and MDM4 (Hoppmann and Wang, 2016). These proteins bind to α-helical protein domains in BID, BIM or p53, respectively, and the groups of Fairlie, Walensky and Wang mimicked these α-helical binding motifs by cyclizing the peptides via ring-closing metathesis. To covalently crosslink the probe to the target protein, the research groups introduced a diaminopropanoic acid residue in the peptide sequence, which was functionalized in the final steps of the synthesis with either an acryl amide or a sulfonyl fluoride. Furthermore, one of the termini of the peptide ligand was equipped with a reporter group that can be used to visualize and enrich the labeling products. The resulting constructs modified their respective targets selectively and two of the probes could be used for in cell fluorescent labeling.

Although synthesis on a solid phase can be considered modular, the number of steps to synthesize the cyclic peptide and to introduce the reactive groups, together with the use of complex building blocks, makes the synthesis of cyclic peptide probes not straightforward. To simplify this process, methods have been developed that allow simultaneous introduction of the reactive group and cyclization of the peptide. Spring and coworkers developed a benzophenone-derivative containing three terminal alkyne groups, one of which was protected with a trimethylsilyl group (compound **17**, Figure 6B). A copper-catalyzed click reaction between the two unprotected alkynes and two azido groups in the peptides of interest yielded stapled α-helical peptides containing the benzophenone photocrosslinker. Subsequent removal of the trimethylsilyl group from the remaining terminal alkyne group liberated the reporter group in the probe, thereby furnishing a fully functional probe (Figure 6B). Spring and coworkers successfully employed this benzophenone derivative for the synthesis of affinity-based probes for MDM2 (Wu et al., 2016).

Li Z. and coworkers also established a method to cyclize peptides and to simultaneously introduce the reactive group (Wang et al., 2019). Starting from linear precursor peptides containing a single cysteine residue and a single methionine residue, they prepared cyclic peptide probes with bifunctional alkylating reagents, such as bis-(bromomethyl)benzene. In this protocol, first the cysteine residue was reacted on the resin with the alkylating reagent to form a stable thioether linkage. Subsequently, the peptide was cleaved from the resin with trifluoroacetic acid, during which spontaneous alkylation of the methionine occurred, thereby cyclizing the peptide (Figure 6C). Interestingly, the formed sulfonium species could act as an electrophilic trap. It was shown that thiols open the sulfonium linkage via a substitution reaction on the linker (Figure 6C). Importantly, the sulfonium was stable when incubated with glutathione for extended time, thus suggesting that the sulfonium group would only undergo reactions with cysteine residues in the proximity of the probe binding pocket. Using this methodology, Li Z. and coworkers designed several cyclic peptides that bind to the PDZ domain of regulator of G-protein 3. The cyclic peptides covalently modified cysteine residues in the binding pocket of the target protein. No reaction was observed when a ligand with a scrambled sequence was used, nor when the two cysteine residues in the vicinity of the protein-peptide interaction site were mutated to serine. The best-binding peptide was then converted into a chemical probe by incorporating a fluorophore on the N-terminus of the cyclic peptide and the resulting probe was tested against the PDZ domain spiked into cell lysate. Promisingly, analysis of the in-gel fluorescence after labeling showed exclusive labeling of the target protein. Thus, the sulfonium tether can be used to generate cyclic peptide probes that can label proteins in a ligand dependent manner. Recently, the same group successfully applied this strategy to prepare affinity-based probes for the abovementioned oncogenic protein BFL-1 (Liu et al., 2021).

A potential downside of this methodology is that alkylation of a methionine residue introduces an additional asymmetric center. As a result, the cyclic peptides are obtained as an epimeric mixture of sulfonium salts. Although the epimers could be separated, the diastereomerically pure sulfonium salts slowly interconverted after purification. Despite the fact that analysis of the peptide epimers with circular dichroism spectroscopy revealed that the sulfonium ion had a limited effect on the three-dimensional structure of the peptide, the stereochemistry of the sulfonium ion might affect the biological activity in some cases.

**Figure 7:**
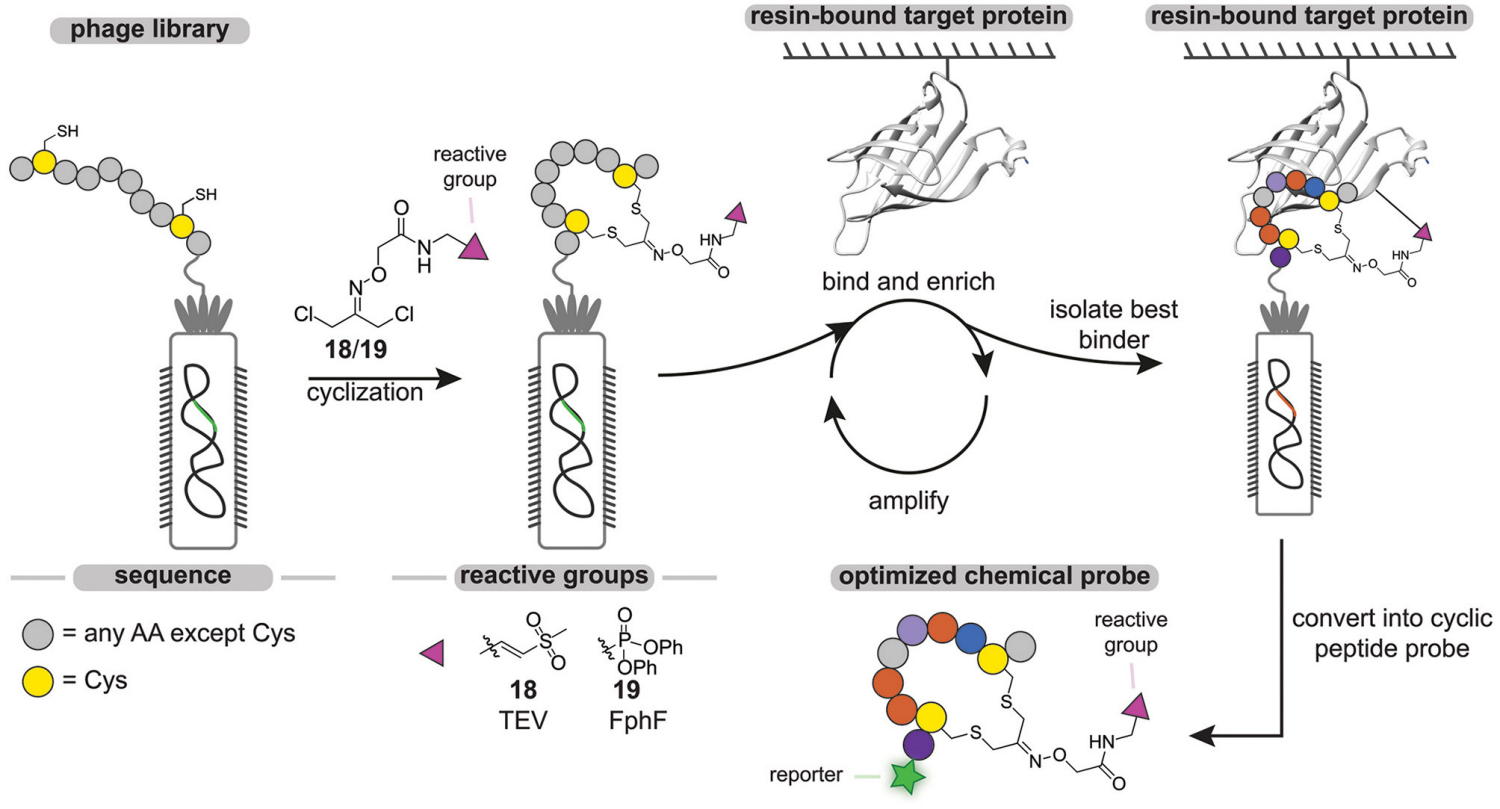
Schematic representation of the phage-display cyclic peptide probe synthesis and screening approach. Different peptides containing two cysteine residues on fixed positions are expressed on the PIII protein of phages. The phage peptide library is cyclized with a dichloroacetone-functionalized vinylsulfone (**18**) or fluorophosphonate (**19**) reactive group. The library is incubated with the immobilized target protein. After the labeling step, all the unbound phage is washed away. The bound phage is liberated by denaturing or by cleavage with a protease. The enriched phage is amplified in *E. coli* cells. After three cycles, the consensus sequence is determined by PCR.

The throughput of chemical probe synthesis with the sulfonium tether is still relatively low, as the peptides have to be synthesized individually. Very recently, Bogyo and coworkers addressed this elegantly (Chen et al., 2020). They exploited phage display technology to generate cyclic peptide-based probes in a modular and high-throughput manner (Figure 7). Phage display, developed in 1985 by Smith (1985), regards the expression of billions of highly diverse peptides on the outer membrane of phages via random mutagenesis (Parmley and Smith, 1988). Cyclic peptides can then be obtained by on-phage cyclization reactions. Much alike DEL, the phage display library is screened against an immobilized target protein and the best binders are isolated by rounds of positive and negative selection. Replication of the isolated phages in a bacterial host and subsequent sequencing enables the identification of consensus sequences of the best binders.

With the aim to develop probes against the Tobacco Etch Virus (TEV) protease and the fluorophosphonate-binding hydrolases F (FphF) from *Staphylococcus aureus*, Bogyo and coworkers prepared a phage library of PIII proteins that express peptides containing two cysteine residues (Chen et al., 2020). TEV protease and FphF belong to the cysteine and serine hydrolase families, respectively. To covalently modify the active site residue of these hydrolases, the peptides were cyclized with a dichloroacetone linker that was functionalized with either a vinyl sulfone (**18**) to target the TEV protease or a diphenyl phosphonate (**19**) warhead to target FphF. Already after three panning rounds, inhibitor consensus sequences could be identified for both proteins. The obtained consensus sequences were readily optimized by mutational studies and yielded low micromolar to high nanomolar inhibitors, demonstrating the power of this approach. The most potent hit for the TEV protease was converted into a probe by introducing a Cy5 fluorophore on the *N*-terminus. Notably, the cyclic peptide chemical probe was able to selectively label the TEV protease in the presence of cell lysate in a ligand-dependent manner, while a linear analog showed prominent labeling of other cysteine proteases.

Overall, introducing a reactive group during the peptide cyclization reaction is an elegant way to develop covalent cyclic peptide inhibitors in a modular manner. By combining it with phage display, it truly becomes a powerful method that is able to screen a wide variety of cyclic peptide probes in a straightforward fashion. Further expansion of the reactive groups that can be used in these approaches will increase the range of proteins that can be targeted. Lastly, in the current approach, the cyclic peptides are converted into chemical probes by addition of a fluorophore only after the best binder has been selected. Although successful in the selected case, reporter groups can have a negative effect on the binding affinity and selectivity of the probe, and the reporter group should therefore preferentially be included in the screening approach. Ideally, one would incorporate a fluorophore or a bioorthogonal tag into the tethering moiety to further simplify the phage display approach.

### Modular Synthesis of Small Molecule Chemical Probes

**Figure 8:**
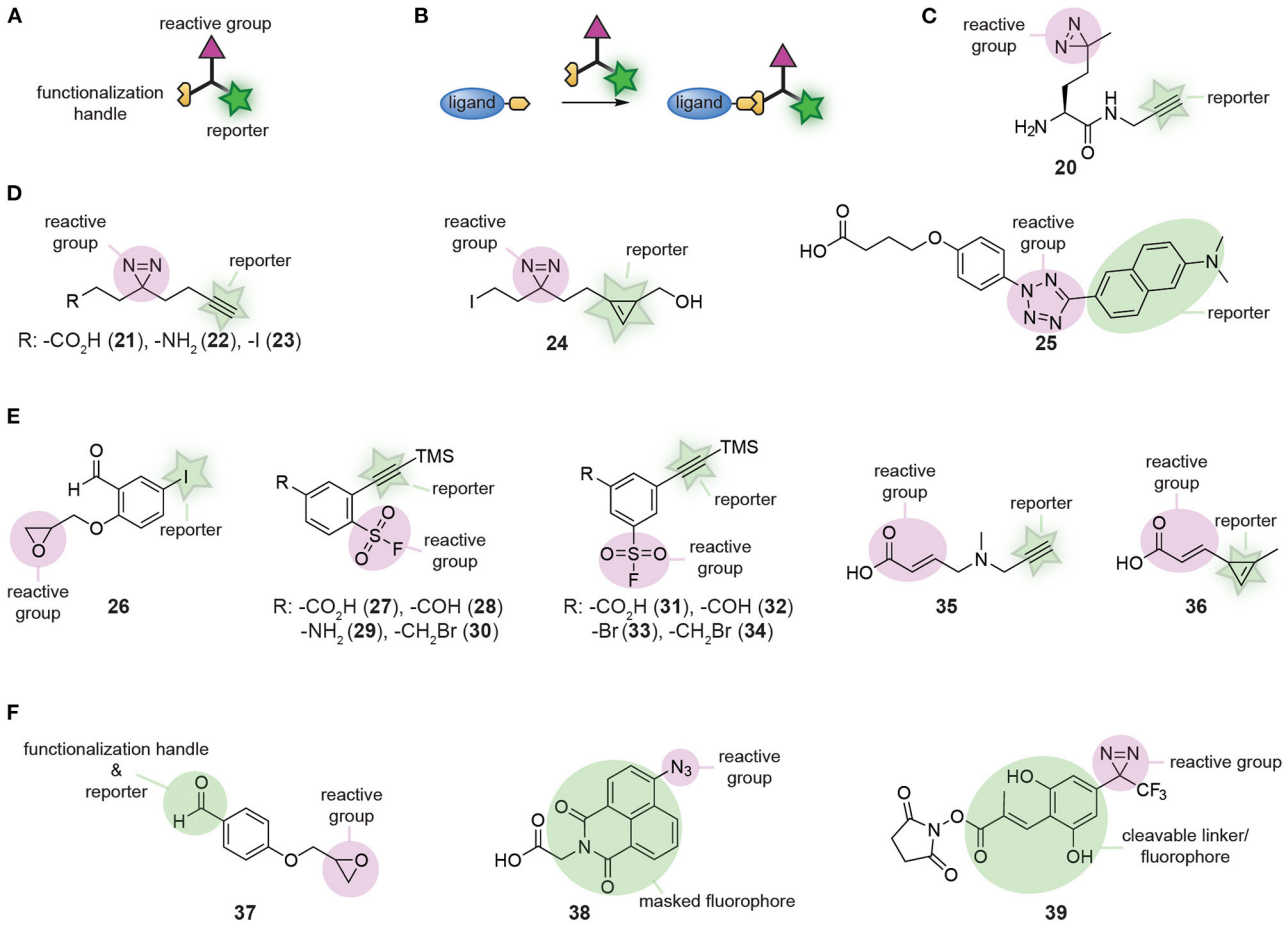
**(A)** Schematic representation of a trifunctional reagent. **(B)** Schematic representation of the synthesis of a probe using a trifunctional reagent. **(C)** Structures of a first-generation trifunctional reagent. **(D)** Examples of minimalistic trifunctional photocrosslinker reagents. **(E)** Examples of minimalistic trifunctional chemical crosslinking reagents. **(F)** Examples of reactive groups that contain dual-purpose elements.

Small molecule affinity-based probes are often prepared by equipping ligands and natural products with a reactive group and a reporter group. Reagents that introduce both groups simultaneously improve the modularity of this process. For this purpose, a wide-variety of trifunctional reagents has been developed. Besides the warhead and the bioorthogonal tag, these molecules contain a third functionality that allows conjugation of the trifunctional reagent to the ligand of interest (Figures 8A,B). The first trifunctional reagents used photocrosslinkers and were prepared starting from amino acids (for an example see **20**, Figure 8C) or via Ugi MCRs (Jacobson et al., 1995; Rowland et al., 2011; Shi et al., 2011; Vinkenborg et al., 2012; Bush et al., 2013). A large variety of ligands has been functionalized with these trifunctional linkers, including kinase inhibitors (Shi et al., 2012), carbohydrates (Sakurai et al., 2014; Yamada et al., 2016), aptamers (Vinkenborg et al., 2012), and even proteins (Hoffmann, 2020). The selectivity and efficacy of the probes formed with these moieties is determined by the linker length and the type of photocrosslinker that is used (Bush et al., 2013; Sakurai et al., 2014, 2015; Kleiner et al., 2017).

The relatively large molecular footprint of these reagents can alter the biological activity of the parent ligand. The crosslinker part may have affinity for proteins and can induce binding to off-targets. This can be suppressed by including a non-targeted control compound, which competes for these crosslinker binding sites (Sakurai et al., 2013). An additional means to reduce the impact of the introduced trifunctional reagent on the binding affinity and selectivity of the probe is by developing minimalistic versions (Li Z. et al., 2013). In these derivatives, the spacer between the crosslinker, the reporter group and the functionalization handle is kept as short as possible. Li Z. et al. developed the first set of minimalistic diazirine photocrosslinkers (Figure 8D). The reported panel contained an alkyne for read-out purposes and either a carboxylic acid (**21**), an amine (**22**), or an alkyl iodide (**23**) to conjugate the reagent to the ligand. Compounds **21** and **22** were employed for amide formation with carboxylic acids and amino groups on the ligand/inhibitor. Alkyl iodide derivative **23** was applied in alkylation reactions. The beneficial effect of the reduced molecular footprint was reflected by the binding affinities of kinase inhibitors equipped with the minimalistic photocrosslinkers. Compared to compounds prepared with amino-acid based photocrosslinkers, the binding affinity of compounds containing **21-23** was closer to that of the parent ligand. Furthermore, labeling of the target was more efficient with these probes. Unfortunately, the alkyne reporter group made **21-23** not suitable for in cell visualization due to the requirement of copper for the click reaction. This limitation was addressed by Li Z. et al. by synthesizing cyclopropene derivative **24**, enabling the detection of target proteins under copper free conditions by an inverse electron-demand Diels-Alder reaction (Li Z. et al., 2014).

Over the course of the past years, numerous other trifunctional molecules have been synthesized, of which each have their own merits. Tetrazole-based photocrosslinker modality **25** was developed by Z. Li et al. as a turn-on fluorescent photocrosslinker that selectively reacts with carboxylic acid residues (Li et al., 2016). Hamachi and colleagues prepared a reactive group that contained an aldehyde, an epoxide and an aryl iodide (**26**, Figure 8E). The aldehyde was used to conjugate the reactive group to a hydrazide-containing ligand. The epoxide provided the reactivity to covalently label the protein of interest. After labeling, the aryl iodide could be further functionalized to introduce a fluorophore or affinity handle via a palladium-catalyzed cross-coupling reaction (Wakabayashi et al., 2008). A diverse panel of clickable sulfonyl fluorides (**27-34**, Figure 8E) that can be used to covalently modify tyrosine and lysine residues in the proximity of the ligand-binding pocket was developed by the group of Robison (Narayanan and Jones, 2015; Fadeyi et al., 2016). Derivatives containing a carboxylic acid, aldehyde, amine, hydroxyl, or alkyl halide functionality were prepared and these reagents could be appended onto ligands with a variety of well-established reactions. Carboxylic acids **27** and **31**, and aldehydes **28** and **32** were conjugated to amino groups of ligands via amide formation and reductive amination, respectively. Benzyl bromides **30** and **34** were employed to alkylate heteroatoms in ligands and aryl halide **33** was used to functionalize ligands via cross-coupling reactions. Recently, a similar set of clickable Michael acceptors (**35** and **36**, Figure 8E) was developed by C. Guo et al. for the selective modification of cysteine residues near the probe binding site (Guo et al., 2019). Using these Michael acceptor modalities, the kinase inhibitors afatinib and ibrutinib were successfully transformed into affinity-based probes for epidermal growth factor receptor (EGFR) and Bruton's tyrosine kinase.

In some cases, one of the functional groups can serve a dual purpose, as was elegantly illustrated by the group of Hamachi (Takaoka et al., 2006). They synthesized a reactive group that contained both an aldehyde and an epoxide group (**37**, Figure 8F). The aldehyde could be used to form probes with hydrazone chemistry and the epoxide enabled covalent modification of the proteins of interest. After labeling, the aldehyde functionality was also employed to visualize the labeled proteins. The reversibility of hydrazone chemistry was exploited by Hamachi and coworkers to exchange the ligand after covalent labeling for an alkoxyamine fluorophore. The reactive group can serve a dual purpose as well, as was exemplified by Singha et al. (2017). They developed a set of 4-azidonaphthalimide photocrosslinker reagents (for an example, see **38**, Figure 8F). UV irradiation of the azido group induced nitrene formation and subsequent reaction with amino acid residues of the target protein. The crosslinked 4-aminonaphthalimide products are fluorescent and thus the 4-azidonaphthalimide served simultaneously as a reactive group and a reporter group. Lastly, Tomohori et al. demonstrated that the introduction of the reporter group may be combined with cleavage of the ligand. They developed *ortho-*hydroxycinnamoyl-based diazirine photocrosslinker reagent **39** (Figure 8F) (Morimoto et al., 2013; Tomohiro et al., 2014). After crosslinking of the probe to the protein of interest, the *E* double bond of the cinnamoyl could be photoisomerized to the *Z* double bond. This isomerization induced lactonization, which resulted in the formation of a fluorescent coumarin derivative and cleavage of the linkage between the ligand and the reactive group. Using one of the groups for a double purpose reduces the molecular footprint of the reagent, thereby minimizing the impact of the crosslinker.

The above-described trifunctional reagents have been employed to develop chemical probes of small molecule ligands or even proteins. The resulting probes have been applied in scenarios ranging from protein labeling in live cells to the proteome profiling of drug molecule targets. One particular application of trifunctional crosslinkers that is worth noting is its use in the identification of the ligandable proteome (Parker et al., 2017). By modifying the small molecular fragments with a trifunctional crosslinker, libraries of covalent fragments have been obtained that were screened in cells or lysates. Mass spectrometry analysis of the modified proteins then revealed the pattern of selectivity for the different fragments and uncovered covalent modifiers of proteins that had not been targeted before.

**Figure 9:**
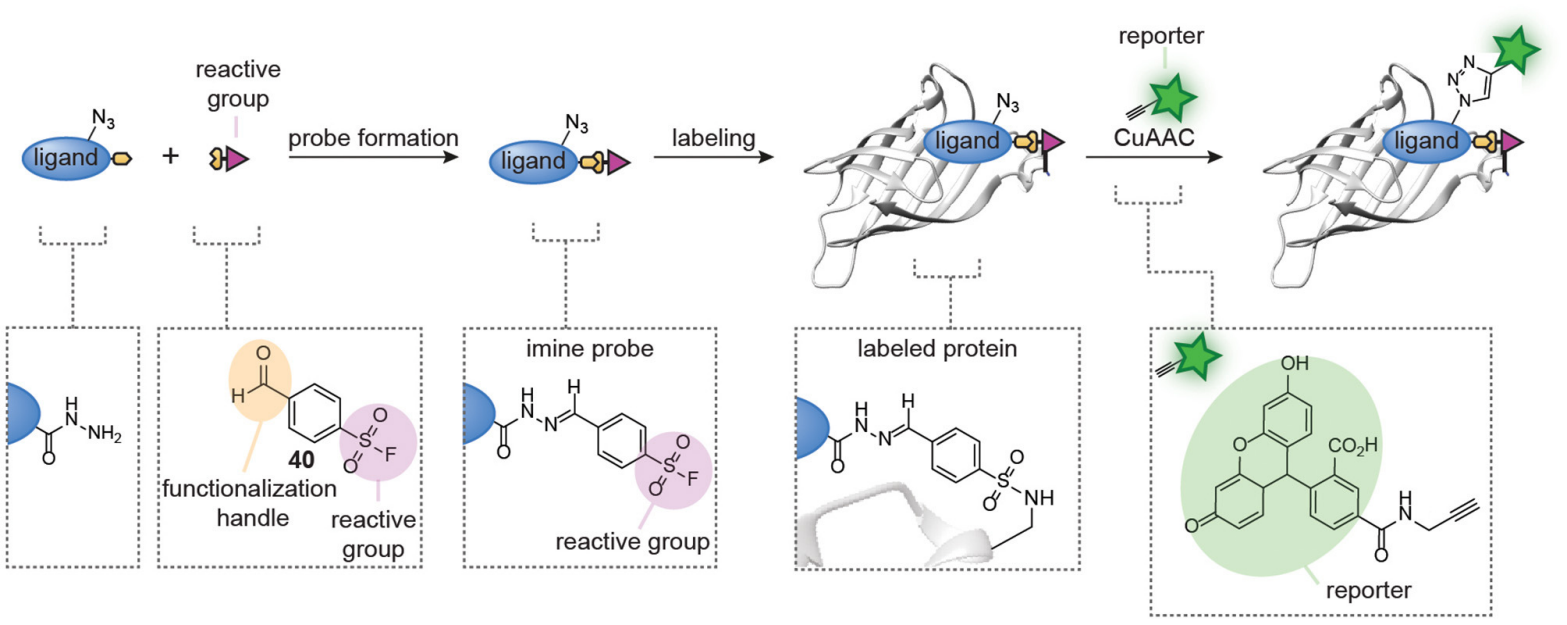
Schematic representation of *in situ* probe formation with hydrazone chemistry. A hydrazide ligand is reacted with an aldehyde-functionalized reactive group, for example a sulfonyl fluoride (**40**), to form a hydrazone probe. The resulting probe is used without further purification. Probe labeling is visualized via a copper-catalyzed click reaction with a reporter group.

Probes formed with trifunctional reagents are generally purified prior to the screening process. Our group reasoned that the possibility to screen reaction mixtures directly should simplify the modular synthesis of small molecule probes further. To enable screening of reaction mixtures, the linking chemistry should be orthogonal to the reactive group chemistry, high-yielding, and fast. Moreover, it should produce a minimal amount of non-invasive side products. Inspired by the fragment-linking and fragment-growing approaches developed in the medicinal chemistry field, in which potent inhibitors are prepared *in situ* from smaller fragments using bioorthogonal reactions, we started to develop similar methods for the synthesis of small molecule probes (Figure 9) (Van Der Zouwen et al., 2019). Hydrazone chemistry was selected for the linking of the probe fragments, as the reaction is bioorthogonal and proceeds cleanly with only water as a byproduct. Furthermore, the pioneering work by the group of Hamachi showed that probes can be prepared by hydrazone formation (Takaoka et al., 2006). Lastly, we hypothesized that hydrazides were straightforward to incorporate into almost any ligand that contains a carboxylic acid or ester group and that the resulting hydrazides could be linked to aldehyde-functionalized reactive groups (see **40** for an example, Figure 9). The clean nature of the hydrazone chemistry should circumvent the need for purification of the probes. As a proof-of-concept, our group prepared a panel of nine different protein reactive groups and we reacted these with biotin-based ligands and sulfonamide-based ligands. With the resulting *in situ* prepared probes, we successfully labeled several biotin-binding proteins and bovine carbonic anhydrase II. Moreover, screening of the probe library on *E. coli* lysate led to the discovery of a novel probe for chloramphenicol acetyl transferase.

**Figure 10:**
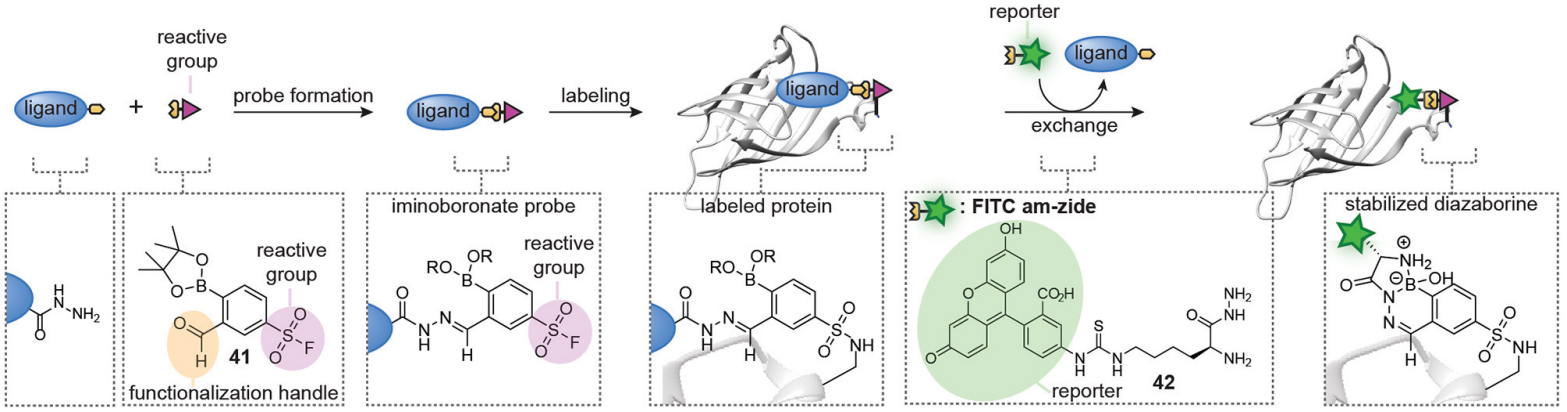
Schematic representation of *in situ* probe formation with iminoboronate chemistry. A hydrazide ligand is reacted with a 2-FPBA-functionalized reactive group, for example a sulfonyl fluoride (**41**), to form an iminoboronate probe. The resulting probe is used without further purification. Probe labeling is visualized via a transimination exchange reaction with an α-amino hydrazide reporter group (**42**). This figure has been derived from van der Zouwen et al. (2020, Figure 1) and is used under a CC-BY 4.0 license.

A limitation of the approach was that exchange of the hydrazone linkage by an alkoxyamine fluorophore was not efficient enough to reliably detect the labeled proteins. While we demonstrated that this limitation can be overcome by introducing a click-handle into the ligand, the additional synthetic steps needed to prepare the required ligands limit the general applicability of the approach. The click handle could also be introduced on the reactive group. We reason that the latter will be more universal than ligand modification, since the same reactive group can be used in different probe development projects and several aldehyde-containing trifunctional reagents are commercially available. However, it does not solve the second limitation of the hydrazone chemistry, which is the slow probe formation. To address both issues simultaneously, our group recently started to explore hydrazone chemistries that allow more facile ligand-fluorophore exchange for *in situ* probe synthesis (Figure 10). We hypothesized that iminoboronate formation between *ortho*-formylphenylboronic acid (2-FPBA) and hydrazides would be particularly suitable for the improved method, since iminoboronates have been shown to be rapidly reversible (Bandyopadhyay and Gao, 2015; Akgun and Hall, 2018; António et al., 2019). Furthermore, the superior reaction rates put this ligation among the fastest bioorthogonal reactions known to date (Schmidt et al., 2015; Gillingham, 2016). Our reasoning indeed proved to be valid (van der Zouwen et al., 2020). Several 2-FPBA-derived reactive groups (see compound **41** for an example, Figure 10) could successfully be coupled to hydrazide ligands and the resulting probes covalently labeled their targets proteins. Transimination with a fluorescein functionalized α-amino hydrazide derivative (FITC am-zide **42**), which had been shown to form stable iminoboronate adducts (Gu et al., 2017), enabled straightforward detection of the labeled proteins. The exchange reaction proceeded efficiently with two to three equivalents of FITC am-zide **42** at pH 5.3 and it reached a plateau after 1–2 h. The exchange also occurred at physiological pH, albeit slower. Importantly, the introduction of the fluorophore moiety did not require the use of a transition metal catalyst. The formation of iminoboronate probes was compatible with several different hydrazide ligands and target proteins, hinting at a general applicability to many different molecular scaffolds. The iminoboronate chemical probes were formed within 15 min after mixing the hydrazide ligands and 2-FPBA-derived reactive groups and could directly be used in protein labeling experiments, without any further purification. This results in a greatly simplified protocol for chemical probe generation and subsequent utilization. Lastly, the probes could even be formed in the context of a protein mixture.

**Figure 11:**
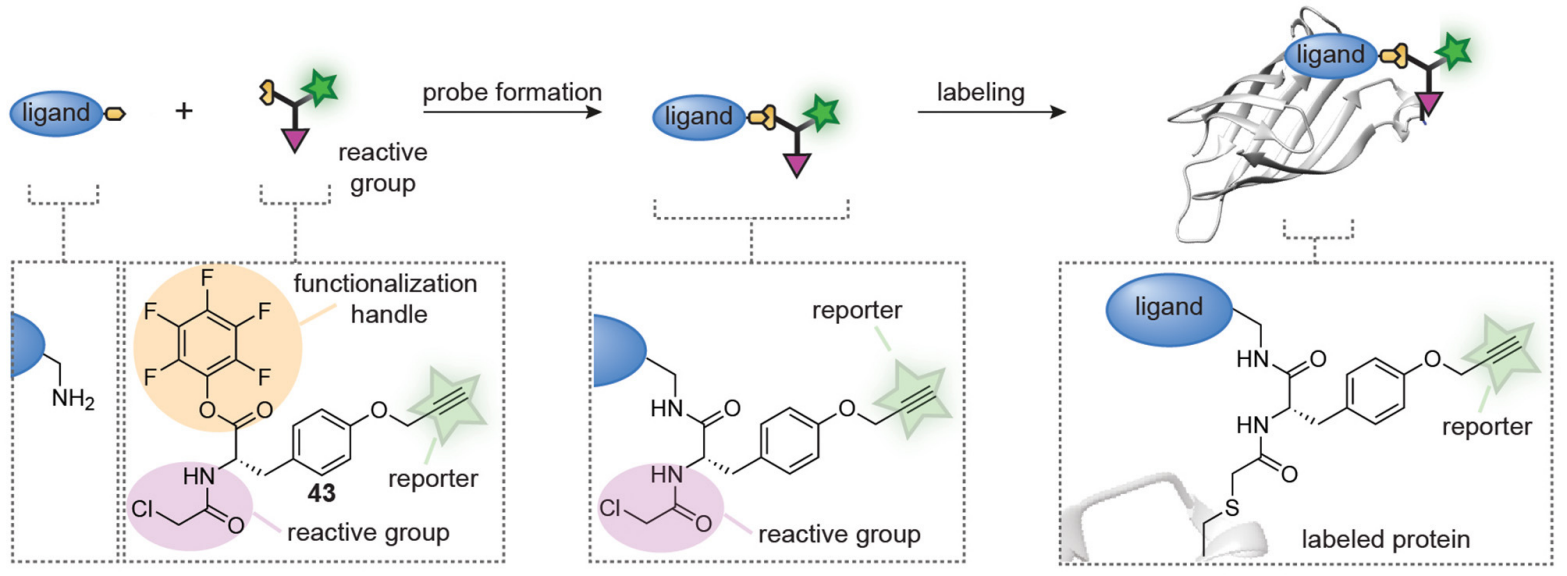
Schematic representation of *in situ* probe formation with a pentafluorophenyl (PFP) ester-functionalized trifunctional reagent (**43**). An amine-containing ligand reacts with the PFP ester to introduce a cysteine reactive chloroacetamide and an alkyne bioorthogonal tag. The resulting probe is used without further purification. Probe labeling is visualized via a copper-catalyzed click reaction with a reporter group.

Very recently, Böttcher et al. also developed a combinatorial approach that allows the in-solution synthesis and screening of probe libraries without purification (Figure 11) (Peñalver et al., 2021). The design of their approach is centered around trifunctional building block **43** that was prepared from tyrosine. The amino group in tyrosine was equipped with a chloroacetamide reactive group to confer reactivity toward cysteine proteases, the phenolic OH was converted into a propargyl ether to allow for visualization of the probe-protein adducts and the carboxylic acid was transformed into a pentafluorophenyl (PFP) ester. The latter part of building block **43** was key to the design. PFP esters react efficiently and cleanly with amino groups to form amide bonds. Moreover, this reaction is readily followed over time by ^19^F-NMR and the group of Böttcher exploited this to optimize the amide coupling. They demonstrated that the trifunctional building block was completely consumed within minutes after the addition of a primary amine. Slower reactions were obtained with aryl amines, but amide formation was still complete after 1 h. After optimization of the coupling conditions, they coupled trifunctional building block **43** to 27 different amines on a microliter scale. Any remaining excess of trifunctional building block **43** was removed by subjecting the reaction mixtures to amino-functionalized polystyrene beads, after which the reaction mixture was lyophilized and used as such. The prepared library was screened against the viral 3C-like protease (3CL^pro^) and papain-like protease (PL^pro^) of SARS-CoV2. As a control, they included mutants in which the active site cysteine is replaced by an alanine residue. Gel-based protein profiling revealed striking differences between the introduced ligands. Both ligands that generated selectivity for 3CL^pro^, as well as ligands that enhanced the selectivity for PL^pro^ were identified from this small library. The resulting probes could be used to label the enzymes in *E. coli* cells and in mammalian cell lysates, and were successfully employed to discover novel inhibitors of these highly relevant cysteine proteases.

In summary, the trifunctional reagents that have been developed up to date have shown to be versatile and widely applicable. The fact that many trifunctional groups are commercially available increases the applicability further. Incorporation of the trifunctional reactive group will be dependent on the availability of a branching point in the ligand that can easily be modified and that is not crucial for binding to the target protein, which could be a potential downside. Probes can be readily prepared by appending different versions of the reported trifunctional reagents onto the scaffold. For immediate screening of reaction mixtures, probes can be prepared in a modular fashion either using hydrazone chemistries or amide bond formation. In particular, iminoboronate chemistry and coupling of PFP-functionalized building blocks have a high potential. The fact that probes can be formed on very small scales within minutes opens opportunities for the rapid synthesis and screening of probe libraries in well-plates. The bioorthogonality of iminoboronate chemistry—probes can even be formed in the presence of proteins—should enable combining hydrazone-based techniques with other modular approaches such as DPAL, DTPC, and phage display. The reversibility of the iminoboronate linker may be used to release the phage, to remove the DNA template or to introduce a reporter group. However, further research will be needed to demonstrate the general applicability, to determine the stability of iminoboronate probes inside cells, and to identify the most suitable α-nucleophiles for probe formation and ligand exchange.

## Discussion

For a long time, combinatorial synthesis of activity- and affinity-based probes was limited to solid phase synthesis. In the past decade, major advances have been made in the development of modular, solution phase synthesis methods. Even though each of these methods aim to prepare fully functional probes from fragments in a limited number of synthetic steps, the strategies that have been developed to link the fragments are very diverse. Each of these solution phase synthesis approaches have their merits and their disadvantages, and which approach to use will depend on the biological questions that are being addressed.

In case the protein target is known, one can imagine that the DNA-templated and phage-display approaches are especially attractive, due to the possibility to screen an enormous number of small molecules and cyclic peptides, respectively. An advantage of the phage display method over DNA-templated chemistries is that it requires minimal synthetic effort. On the other hand, phage-display is limited to peptides, while the scaffolds that can be used in the DNA-templated approaches are much more diverse. Moreover, it has been shown that the DNA-templated approaches can be used for screening in mammalian cells. Both methods also have their limitations. They require that the protein target can be stably expressed and immobilized or enriched. Furthermore, without a cell-penetrating peptide, DNA-templated probes are poorly taken up by cells, which limits their usefulness for cellular applications, and therefore DNA-templated probes should be converted to a small molecule probe. Therefore, future work should be directed toward developing methods that can be used to transform a DNA-templated probe into a small molecule probe. The fragment linking strategies developed in the medicinal chemistry field may be used to achieve this goal. A limitation of the phage-display probe synthesis approach is that it for now only has been used in the development of activity-based probes. In order to also access affinity-based probes, future research should therefore be directed toward adapting the phage-display approach to affinity-based probes for example by combining with the sulfonium-cyclization, or with the trifunctional reagents and dual-purpose reagents.

In case the protein targets are unknown and the ligand is taken as the starting point, then the trifunctional and dual-purpose reagents seem most desirable, although DNA-templated approaches also may be used. Ideally, the trifunctional and dual-purpose reagents will be compiled into a probe-development kit that is available to the community. Key to the success will be the development of simple and robust conjugation strategies for the introduction of the trifunctional reagent on the scaffold of interest, as well as the addition of new reactive group chemotypes. Preferably, the resulting probes can be screened without purification. Robust conjugation chemistries will allow labs that have limited experience with organic chemistry to prepare probes for the protein of interest. Unfortunately, which part of the ligand scaffold is most suited to introduce the minimal reactive groups will still be determined through trial-and-error.

Finally, the MCRs and SuFEx click reactions seem most suited in case one wants to quickly generate a large library for chemoproteomic profiling. These approaches might be further supplemented by the use of trifunctional and dual-purpose reagents as well. Reactions that introduce the reactive groups into libraries and allow subsequent screening without intermediate purification, like the SuFEx click and the iminoboronate formation, might be particularly attractive as they should be more compatible with larger libraries.

In conclusion, modular synthesis of probes is rapidly evolving field. Many interesting new approaches have been developed over the past years and it is foreseen that these strategies will lead to the discovery of novel chemical probes. The techniques will continue to evolve and this will likely lead to more strategies that can be used to screen probe mixtures directly.

## Author Contributions

AZ and MW have written the manuscript and have prepared the figures. Both authors contributed to the article and approved the submitted version.

## Conflict of Interest

The authors declare that the research was conducted in the absence of any commercial or financial relationships that could be construed as a potential conflict of interest.
